# Dynamic risk prediction for diabetes using biomarker change measurements

**DOI:** 10.1186/s12874-019-0812-y

**Published:** 2019-08-14

**Authors:** Layla Parast, Megan Mathews, Mark W. Friedberg

**Affiliations:** 1 RAND Corporation, 1776 Main St, Santa Monica, CA 90401 USA; 2 RAND Corporation, 20 Park Plaza # 920, Boston, MA 02116 USA

**Keywords:** Diabetes, Prediction, Statistical methods

## Abstract

**Background:**

Dynamic risk models, which incorporate disease-free survival and repeated measurements over time, might yield more accurate predictions of future health status compared to static models. The objective of this study was to develop and apply a dynamic prediction model to estimate the risk of developing type 2 diabetes mellitus.

**Methods:**

Both a static prediction model and a dynamic landmark model were used to provide predictions of a 2-year horizon time for diabetes-free survival, updated at 1, 2, and 3 years post-baseline i.e., predicting diabetes-free survival to 2 years and predicting diabetes-free survival to 3 years, 4 years, and 5 years post-baseline, given the patient already survived past 1 year, 2 years, and 3 years post-baseline, respectively. Prediction accuracy was evaluated at each time point using robust non-parametric procedures. Data from 2057 participants of the Diabetes Prevention Program (DPP) study (1027 in metformin arm, 1030 in placebo arm) were analyzed.

**Results:**

The dynamic landmark model demonstrated good prediction accuracy with area under curve (AUC) estimates ranging from 0.645 to 0.752 and Brier Score estimates ranging from 0.088 to 0.135. Relative to a static risk model, the dynamic landmark model did not significantly differ in terms of AUC but had significantly lower (i.e., better) Brier Score estimates for predictions at 1, 2, and 3 years (e.g. 0.167 versus 0.099; difference − 0.068 95% CI − 0.083 to − 0.053, at 3 years in placebo group) post-baseline.

**Conclusions:**

Dynamic prediction models based on longitudinal, repeated risk factor measurements have the potential to improve the accuracy of future health status predictions.

**Electronic supplementary material:**

The online version of this article (10.1186/s12874-019-0812-y) contains supplementary material, which is available to authorized users.

## Background

In recent years, a wide range of markers have become available as potential tools to predict risk or progression of disease, leading to an influx of investment in the area of personalized screening, risk prediction, and treatment [1–4]. However, many of the available methods for personalized risk prediction are based on snapshot measurements (e.g., biomarker values at age 50) of risk factors that can change over time, rather than longitudinal sequences of risk factor measurements [2, 5–7]. For example, the Framingham Risk Score estimates the 10-year risk of developing coronary heart disease as a function of *most recent* diabetes status, smoking status, treated and untreated systolic blood pressure, total cholesterol, and HDL cholesterol [6]. With electronic health record and registry data, incorporating *repeated* measurements over a patient’s longitudinal clinical history, including the trajectory of risk factor changes, into risk prediction models is becoming more realistic and might enable improvements upon currently-available static prediction approaches [8, 9].

Specifically considering prediction of incident type 2 diabetes, a recent systematic review by Collins et al. [10] found that the majority of risk prediction models have focused on risk predictors assessed at a fixed time; the most commonly assessed risk predictors were age, family history of diabetes, body mass index, hypertension, waist circumference and gender. For example, Kahn et al. [11] developed and validated a risk-scoring system for 10-year incidence of diabetes including (but not limited to) hypertension, waist circumference, weight, glucose level, and triglyceride level using clinical data from 9587 individuals. Models that aim to incorporate the trajectory of risk factor changes, e.g., the *change* in a patient’s glucose level in the past year, into risk prediction for incident diabetes have been sparse. Some available methods that allow for the use of such longitudinal measurements are often considered overly complex or undesirable due to restrictive parametric modeling assumptions or infeasible due to computational requirements [12–15]. That is, with these methods it is often necessary to specify a parametric model for the longitudinal measurements, and a parametric or semiparametric model characterizing the relationship between the time-to-event outcome and the longitudinal measurements and then use, for example, a Bayesian framework to obtain parameter estimates.

Recently, the introduction of the dynamic landmark prediction framework has proved a useful straightforward alternative in several other clinical settings [16–19]. In the dynamic prediction framework, the risk prediction model for the outcome of interest is updated over time at pre-specified “landmark” times (e.g. 1 year or 2 years after the initiation of a particular medication) incorporating information about the change in risk factors up to that particular time. That is, suppose the goal is to provide an individual with the predicted probability of survival past time *τ* = *t* + *t*_0_ given that he/she has already survived to time *t*_0_ (*t*_0_ is the landmark time), the dynamic prediction approach provides this prediction using a model that is updated at time *t*_0_ such that it can incorporate the information available up to time *t*_0_. The approach is appealing because it is relatively simple and straightforward, and does not require as strict parametric modeling assumptions as is required by a joint modeling approach.

In this paper, we describe the development and use of a dynamic prediction model to estimate the risk of developing type 2 diabetes mellitus, incorporating biomarker values measured repeatedly over time, using data from the Diabetes Prevention Program study. We compare our dynamic prediction approach to a static prediction model to determine whether improvements in prediction accuracy can be obtained. Our aim is to illustrate how such a dynamic approach may be useful and appealing to both clinicians and patients when developing prediction models for the incidence of type 2 diabetes.

## Methods

### Static prediction model

For each individual *i*, let *Z*_*i*_ denote the vector of available baseline covariates, *T*_*i*_ denote the time of the outcome of interest, *C*_*i*_ denote the censoring time assumed to be independent of *T*_*i*_ given *Z*_*i*_, *X*_*i*_ = min(*T*_*i*_, *C*_*i*_) denote the observed event time, and *D*_*i*_ = *I*(*T*_*i*_ < *C*_*i*_) indicate whether the event time or censoring time was observed. Suppose the goal is to predict survival to some time *τ* for each individual *i*, based on their covariates *Z*_*i*_. A static model based on the Cox proportional hazards model [20, 21] can be expressed as:

1.1 $$ P\left({T}_i>\tau |{Z}_i\right)=\exp \left\{-{\varLambda}_0\left(\tau \right)\mathit{\exp}\left({\beta}^{\prime }{Z}_i\right)\right\} $$

in terms of survival past time t, or in terms of the hazard function as

1.2 $$ \lambda \left(\tau |{Z}_i\right)={\uplambda}_0\left(\tau \right)\ e\mathrm{x}p\left({\beta}^{\prime }{Z}_i\right) $$

where *Λ*_0_(*τ*) is the cumulative baseline hazard at time *τ*, λ_0_(*τ*) is the baseline hazard at time *τ*, and *β* is the vector of regression parameters to be estimated. Estimates of *β* are obtained by maximizing the partial likelihood [22].

Here, we use the term “static” because the *model* itself never changes; the model is fit once, the *β* vector of parameters is estimated, and these estimates are used to calculate an individual’s predicted probability of survival given their particular *Z*_*i*_. In practice, even when *Z*_*i*_ is actually a vector of covariate values measured after baseline (e.g. 1 year later), this model is still used under this static approach. This type of model is standard in the risk prediction literature [2, 6, 7, 10, 23]. For example, with the Framingham risk score, there is a single static model that is used to provide risk estimates to patients – whether a patient comes in at age 40 or age 60 (using age as the time scale), the actual *β* estimates used to calculate risk are the same, only the *Z*_*i*_ values potentially change to reflect the current covariates values.

### Dynamic prediction model

A dynamic prediction model differs from a static prediction model in that the *model itself* is updated (i.e., refit) at specified “landmark times” e.g. 1 year, 2 years, 3 years after baseline [17, 18, 24]. This model can be expressed as a landmark Cox proportional hazards model:

1.3 $$ P\left({T}_i>\tau |{T}_i>{t}_0,{Z}_i\left({t}_0\right)\right)=\exp \left\{-{\varLambda}_0\left(\tau |{t}_0\right)\mathit{\exp}\left({\alpha}^{\prime }{Z}_i\left({t}_0\right)\right)\right\} $$

in terms of survival past time *τ*, or in terms of the hazard function as

1.4 $$ \lambda \left(\tau |{t}_0,{Z}_i\left({t}_0\right)\right)={\uplambda}_0\left(\tau |{\mathrm{t}}_0\right)\ \mathit{\exp}\left({\alpha}^{\prime }{Z}_i\left({t}_0\right)\right) $$

where *t*_0_ is the landmark time, *τ* = *t* + *t*_0_, *t* is referred to as the “horizon time”, *Z*_*i*_(*t*_0_) denotes a vector of covariates and (if available) covariates that reflect changes in biomarker values from baseline to *t*_0_, *Λ*_0_(*τ*| *t*_0_) is the cumulative baseline hazard at time *τ* given survival to t_0_, λ_0_(*τ*| t_0_) is the baseline hazard at time *τ* given survival to t_0_, and *α* is the vector of regression parameters to be estimated at each time t_0_. As in model (1.1), estimates of *α* are obtained by maximizing the appropriate partial likelihood. However, for estimation of *α*, model (1.3) is fit only among individuals surviving to t_0_ and thus, the partial likelihood is composed of only these individuals.

The key substantive differences between the static and dynamic landmark models are that (1) no information regarding *change* in covariate (e.g., biomarker) measurements are incorporated in the static approach, (2) no information regarding survival up to t_0_ is incorporated in the static approach, and (3) the static approach uses a single model (i.e. a single set of Cox regression coefficients) for all predictions, whereas the dynamic landmark model fits an updated model at each landmark time and thus, has a distinct set of regression coefficients for each t_0_. Importantly, the probability being estimated with the static model vs. the landmark model is different and thus, the resulting interpretation of this probability is different between the two approaches. The static model estimates *P*(*T*_*i*_ > *τ*| *Z*_*i*_), ignoring any information about survival to *t*_0_ while the landmark model estimates *P*(*T*_*i*_ > *τ*| *T*_*i*_ > *t*_0_, *Z*_*i*_(*t*_0_)), explicitly incorporating information about survival to *t*_0_ and changes in biomarker values from baseline to *t*_0_. Of course, a simple derivation can be used to show that one could obtain an estimate for *P*(*T*_*i*_ > *τ*| *T*_*i*_ > *t*_0_, *Z*_*i*_) using the static model based on model (1.1) as $\exp \left\{-\left({\hat{\varLambda}}_0\left(\tau \right)-{\hat{\varLambda}}_0\left({t}_0\right)\right)\mathit{\exp}\left({\hat{\beta}}^{\prime }{Z}_i\right)\right\}$ where $\hat{\beta}$ and ${\hat{\varLambda}}_0$ denote the estimates of the regression coefficients from maximizing the partial likelihood and the Breslow estimator of the baseline cumulative hazard, respectively. However, this is not what is done in current practice when using a static model; the estimated *P*(*T*_*i*_ > *τ*| *Z*_*i*_) is typically provided to patients even when it is known they have survived to *t*_0_ e.g. the patient is given this prediction at a 1 year post-intervention appointment time, *t*_0_ = 1 year. In addition, even with this calculation, the estimation of $\hat{\beta}$ and ${\hat{\varLambda}}_0$ themselves are not restricted to individuals that survive to *t*_0_ but were instead estimated using all patients at baseline.

Using the dynamic prediction model, one would generally expect improved prediction accuracy due to the fact that the updated models are taking into account survival to t_0_ and should more precisely estimate risk for patients after time t_0_. Indeed, previous work has shown, through simulations and applications outside of diabetes, the benefits of this dynamic approach compared to a static model [24]. Parast & Cai [24] demonstrated through a simulation study improved prediction performance when a dynamic landmark prediction model was used instead of a static model in a survival setting.

With respect to the selection of the times t_0_, these times are generally chosen based on the desired prediction times relevant to the particular clinical application. For example, if patients come in for yearly appointments, the t_0_ times of interest may be 1 year, 2 years, and 3 years. If patients come in every 2 years, the t_0_ times of interest may be 2 years and 4 years.

### Model assumptions and model complexity

Both the static model and dynamic prediction model described above rely on correct specification of the relevant models (models (1.2) and (1.4), respectively). Correct model specification includes the assumption of linearity in the covariates (i.e., *β*^′^*Z*_*i*_), the assumption of no omitted confounders, and the proportional hazards assumption. The proportional hazards assumption states that the ratio of the hazards for two different individuals is constant over time; this can be seen in the specification of model (1.2) where the hazard ratio for two individuals *λ*(*τ*| *Z*_*i*_) and *λ*(*τ*| *Z*_*j*_) can be seen to be *exp*(*β*^′^(*Z*_*i*_ − *Z*_*j*_)) which is not a function of time. The simulation study of Parast & Cai [24] showed that when model (1.2) holds, the static model and dynamic landmark model perform equally well, but when this model is not correctly specified, the dynamic landmark model outperforms the static model.

Models (1.2) and (1.4) are relatively straightforward. These models could certainly be altered to incorporate desired complexities including more complex functions of the covariates, spline or other basis expansions, and/or regularized regression. In addition, this dynamic prediction framework is not restricted to the Cox proportional hazards model alone. Other modeling approaches appropriate for time-to-event outcome can be considered here including an accelerated failure time model, proportional odds model, or even a fully non-parametric model if there are only 1–2 covariates and the sample size is very large [25, 26].

### Evaluation of prediction accuracy

To evaluate the accuracy of the prediction models in this paper, we assessed both discrimination and calibration. Discrimination measures the extent to which the prediction rule can correctly distinguish between those who will be diagnosed with diabetes within 2 years and those who will not. As a measure of discrimination, we used the area under the receiver operating characteristic curve (AUC) [27, 28] defined as:

$$ {AUC}_K\left(\tau, {\mathrm{t}}_0\right)=P\left({\hat{\mathrm{p}}}_{Ki}<{\hat{\mathrm{p}}}_{Kj}\right|{\mathrm{t}}_0<{T}_i\le \tau, {T}_j>\tau \Big) $$

for K = D, S (i.e., dynamic and static), where ${\hat{\mathrm{p}}}_{Di}$ and ${\hat{\mathrm{p}}}_{Si}$ indicate the predicted probability of survival to time *τ* using the dynamic model and static model, respectively, for person *i*. The AUC ranges from 0 to 1 with higher values indicating better prediction accuracy. The AUC has an appealing interpretation as the probability that the prediction model being evaluated will assign a *lower* probability of survival to an individual that will actually experience the event within the time period of interest, compared to an individual that will not.

Calibration is based on the alignment between observed event-rates and predicted event probabilities (i.e., how well predictions match observed rates). As a measure of calibration, we used the Brier Score [29, 30] defined as:

$$ {BS}_K\left(\tau, {\mathrm{t}}_0\right)=E\left({\left[I\left({T}_i>\tau \Big)-{\hat{\mathrm{p}}}_{Ki}\right|{T}_i>{\mathrm{t}}_0\right]}^2\right) $$

for K = D, S. The Brier Score ranges from 0 to 1 with lower values indicating better prediction accuracy. The Brier Score captures the mean squared error comparing the true event rates and the predicted event rates obtained from the prediction model. As a test of calibration, we additionally calculated the Hosmer-Lemeshow goodness of fit test statistic (extended to survival data) [31, 32]. We compare the AUC, Brier Score, and Hosmer-Lemeshow test statistic from the dynamic model versus the static model.

Lastly, as another measure of comparison between the dynamic and static model, we calculated the net reclassification improvement (NRI) [33, 34]. The NRI quantifies how well a new model (the dynamic model) reclassifies individuals in terms of estimated risk predictions, either appropriately or inappropriately, as compared to an old model (the static model).

For all AUC, Brier Score and NRI, we used a nonparametric inverse probability of censoring weighted estimation approach that does not rely on the correct specification of any of the prediction models described above [28, 35] and bootstrapped the approach using 500 samples to obtain confidence intervals and *p*-values [36]. In addition, for all four accuracy metrics, we used general cross-validation whereby we repeatedly split the data into a training set and a test set during the estimation process to guard against over-fitting (as we did not have access to an external validation data source) [37, 38]. That is, when the same dataset is used to both construct a prediction rule and evaluate a prediction rule, the prediction accuracy measures can sometimes appear overly optimistic because the prediction rule has been over-fit on the single dataset available. Therefore, the accuracy observed may not reflect what one could expect to see using an external validation data source. Cross-validation is helpful in settings where only one dataset is available; data are split such that some portion is used to “train” the prediction rule (build the model) and the remainder is used to “test” the prediction rule i.e., evaluate the accuracy. This is not as ideal as having access to an external validation source, but is more beneficial than no cross-validation at all. For our analysis, we took a random sample of 2/3 of the data to use as a training set, and the remaining 1/3 of the data was the test set. This random splitting, fitting, and evaluating, was repeated 100 times and the average of those 100 estimates was calculated.

### Application to diabetes prevention program: study description

Details of the Diabetes Prevention Program (DPP) have been published previously [39, 40]. The DPP was a randomized clinical trial designed to investigate the efficacy of multiple approaches to prevent type 2 diabetes in high-risk adults. Enrollment began in 1996 and participants were followed through 2001. Participants were randomly assigned to one of four groups: metformin (*N* = 1073), troglitazone (*N* = 585; this arm was discontinued due to medication toxicity), lifestyle intervention (*N* = 1079) or placebo (*N* = 1082). After randomization, participants attended comprehensive baseline and annual assessments as well as briefer quarterly visits with study personnel. In this paper, we focus on the placebo and metformin groups. Though lifestyle intervention was found to be more effective in terms of reducing diabetes incidence in the main study findings [40], prescribing metformin for patients at high-risk of diabetes is becoming more common in current clinical practice and thus, this comparison is likely of more practical interest [41]. We obtained data on 2057 DPP participants (1027 in metformin arm, 1030 in placebo arm) collected on or before July 31, 2001 as part of the 2008 DPP Full Scale Data Release through the National Institute of Diabetes and Digestive and Kidney Diseases (NIDDK) Data Repository, supplemented by participant data released by the 2011 Diabetes Prevention Program Outcomes Study, which followed participants after the conclusion of DPP, through August 2008. The median follow-up time in this cohort was 6.11 years.

The primary outcome was time to development of type 2 diabetes mellitus, measured at mid-year and annual study visits, as defined by the DPP protocol: fasting glucose greater than or equal to 140 mg/dL for visits through 6/23/1997, greater than or equal to 126 mg/dL for visits on or after 6/24/1997, or 2-h post challenge glucose greater than or equal to 200 mg/dL. For individuals who did not develop type 2 diabetes mellitus, their observation time was censored on the date of their last visit within the study.

Available patient non-laboratory baseline characteristics included age group (< 40, 40–44, 45–49, 50–54, 55–59, 60–64, 65+), gender, body mass index group (BMI; < 30 kg/m^2^, ≥30 to < 35 kg/m^2^, ≥35 kg/m^2^), smoking status (yes, no, not available), and race/ethnicity (White, Black, Hispanic, Other). These variable aggregations, which result in some information loss, were instituted in the NIDDK data release to protect patient confidentiality. Laboratory values included fasting plasma glucose and hemoglobin A1c (HbA1c) measured at randomization (i.e., baseline), at 6 months post-randomization, and at annual visits thereafter. For each laboratory measurement after baseline, we calculated change-from-baseline values for use in our prediction models.

This study (a secondary data analysis) was approved by RAND’s Human Subjects Protection Committee.

### Application to diabetes prevention program: analysis

In this application, our goal was to provide predictions of a 2-year horizon time for diabetes-free survival, updated at 1, 2, and 3 years post-baseline. That is, we are predicting diabetes-free survival to 2 years post-baseline, and then predicting diabetes-free survival to 3 years, 4 years, and 5 years post-baseline, given the patient already survived to 1 year, 2 years, and 3 years post-baseline, respectively. In our defined notation, *τ* = 2, 3, 4, 5 years and *t*_0_ = 0, 1, 2, 3 years and *t* = 2 years. Our focus on somewhat short-term survival here is due to both data availability for this study and the fact that the study population is composed of high-risk individuals.

We first fit the static model (model (1.2)) with covariates age, gender, BMI, smoking status, race/ethnicity, and baseline (the time of randomization) measurements of HbA1c and fasting plasma glucose. Recall that this results in a single model, with a single set of regression coefficients. To obtain our predictions of interest from the static model when *t*_0_ > 0, probabilities were calculated using the HbA1c and fasting plasma glucose measurements at *t*_0_, applied to this single model.

Next, we fit dynamic landmark prediction models where we additionally incorporate information on survival to the landmark times *t*_0_ = 1, 2, 3 years and information on the change in HbA1c and fasting plasma glucose from baseline to *t*_0_. These models result in an estimate of the probability of a diabetes diagnosis within 2 years after the landmark time as a function of baseline characteristics, lab measurements at baseline, and the *change* in lab measurements from baseline to *t*_0_. This approach results in four models, each with its own set of regression coefficients. (Note that at baseline, the static model is equivalent to the dynamic model.) The full dynamic model framework thus results in estimates of: (a) a patient’s 2-year predicted probability of developing diabetes at baseline (*t*_0_ =0; same as static model), (b) an *updated* 2-year predicted probability for a patient at the landmark time (t_0_ = 1 year), for patients who survived 1 year after baseline without a diabetes diagnosis, incorporating both the change in laboratory values and the patient’s diabetes-free survival over the last year, (c) a similarly updated 2-year prediction at 2 years post-baseline, (d) a similarly updated 2-year prediction at 3 years post-baseline.

We stratified all analyses by treatment group: placebo and metformin.

### Data availability, code and software

DPP data are publicly available upon request from the NIDDK Data Repository and require the establishment of a data use agreement. Code for all analyses presented here is available upon request from the authors. All analyses were performed in R Version 3.3.2, an open source statistical software, using the packages survival and landpred.

## Results

Approximately 49% of participants in our sample were younger than 50, 67% were female, and the majority were of white race (Table 1). At baseline, more than one-third of participants had BMI greater than 35 kg/m^2^, and the majority did not smoke. Previous analyses have shown that these characteristics were balanced across the randomized treatment groups [40, 42]. Eight participants were missing HbA1c values at baseline and were thus excluded from our subsequent analyses.

**Table 1:** Baseline characteristics of analytic sample

	Overall (*N* = 2057) N(%) or Mean (SD)	Placebo (*N* = 1027) N(%) or Mean (SD)	Metformin (*N* = 1030) N(%) or Mean (SD)
Age			
< 40	286 (13.9%)	151 (14.7%)	135 (13.1%)
40–44	306 (14.9%)	147 (14.3%)	159 (15.5%)
45–49	422 (20.5%)	231 (22.4%)	191 (18.6%)
50–54	376 (18.3%)	167 (16.2%)	209 (20.4%)
55–59	255 (12.4%)	134 (13%)	121 (11.8%)
60–64	201 (9.8%)	100 (9.7%)	101 (9.8%)
65+	211 (10.3%)	100 (9.7%)	111 (10.8%)
Gender			
Male	689 (33.5%)	174 (33.8%)	186 (36.3%)
Female	1368 (66.5%)	699 (67.9%)	669 (65.1%)
BMI			
< 30 kg/m^2^	665 (32.3%)	326 (31.7%)	339 (33%)
≥ 30 to < 35 kg/m^2^	620 (30.1%)	297 (28.8%)	323 (31.5%)
≥ 35 kg/m^2^	772 (37.5%)	407 (39.5%)	365 (35.5%)
Smoking Status			
Yes	136 (6.6%)	71 (6.9%)	65 (6.3%)
No	1764 (85.8%)	878 (85.2%)	886 (86.3%)
Not available	157 (7.6%)	81 (7.9%)	76 (7.4%)
Race/ethnicity			
White	1188 (57.8%)	586 (56.9%)	602 (58.6%)
Black	440 (21.4%)	219 (21.3%)	221 (21.5%)
Hispanic	330 (16%)	168 (16.3%)	162 (15.8%)
Other	99 (4.8%)	57 (5.5%)	42 (4.1%)
Fasting plasma glucose (mg/dL)	107.35 (7.84)	107.42 (7.83)	107.27 (7.86)
Hemoglobin A1c (%)^a^	5.91 (0.51)	5.91 (0.5)	5.91 (0.51)

^a^ Eight participants were missing HbA1c, calculation is among non-missing values

A total of 182 participants assigned to the placebo arm (18%) and 126 participants assigned to the metformin arm (12%) were diagnosed with diabetes within 2 years of baseline. Among the 866 placebo participants and 914 metformin participants who survived to 1 year post-baseline without a diabetes diagnosis, 159 (18%) and 140 (15%) were diagnosed with diabetes within 2 years (i.e., by 3 years post-baseline), respectively. Among the 748 placebo participants and 815 metformin participants who survived to 2 years without a diabetes diagnosis, 105 (14%) and 127 (16%) were diagnosed with diabetes within 2 years (i.e., by 4 years post-baseline), respectively. Among the 638 placebo participants and 703 metformin participants who survived to 3 years without a diabetes diagnosis, 73 (11%) and 74 (11%) were diagnosed with diabetes within 2 years (i.e., by 5 years post-baseline), respectively.

In the baseline static prediction model for the placebo arm, the risk of developing diabetes within 2 years was higher for BMI ≥35 kg/m^2^ than for BMI < 30 kg/m^2^ (hazard ratio [HR] = 1.28, *p* < 0.05) and higher among Hispanic than among white participants (HR = 1.31, *p* < 0.05) (Table 2). In both treatment arms, higher baseline fasting plasma glucose and HbA1c were associated with higher diabetes risk (for glucose, HR = 1.08 in the placebo arm and 1.05 in the metformin arm, *p* < 0.001; for HbA1c, HR =1.52 and 1.73, *p* < 0.001). In the dynamic models (see Additional file 1 for model results), the risks associated with each variable changed over time and as expected, larger changes (increases) in fasting plasma glucose and HbA1c compared to baseline were associated with higher diabetes risk.

**Table 2:** Static prediction model

	Placebo Hazard Ratio (95% Confidence Interval)	Metformin Hazard Ratio (95% Confidence Interval)
Age		
< 40	REF	REF
40–44	1.17 (0.84,1.63)	1.05 (0.72,1.52)
45–49	1.07 (0.79,1.45)	0.93 (0.65,1.34)
50–54	0.9 (0.64,1.25)	0.95 (0.67,1.34)
55–59	0.76 (0.53,1.1)	0.8 (0.53,1.21)
60–64	0.91 (0.61,1.36)	1.07 (0.72,1.6)
65+	0.98 (0.64,1.49)	1 (0.66,1.51)
Gender		
Male	REF	REF
Female	1.04 (0.85,1.28)	1.14 (0.92,1.42)
BMI		
< 30 kg/m^2^	REF	REF
≥ 30 to < 35 kg/m^2^	0.96 (0.75,1.22)	0.91 (0.71,1.18)
≥ 35 kg/m^2^	1.28 (1.02,1.62)*	1 (0.78,1.29)
Smoking Status		
Yes	0.93 (0.67,1.3)	1.33 (0.91,1.94)
No	REF	REF
Not available	1.15 (0.82,1.62)	1.31 (0.92,1.87)
Race/ethnicity		
White	REF	REF
Black	1.13 (0.89,1.43)	0.94 (0.73,1.22)
Hispanic	1.31 (1,1.7)*	0.98 (0.74,1.3)
Other	1.34 (0.89,2.01)	0.86 (0.5,1.47)
Fasting plasma glucose (mg/dL)	1.08 (1.07,1.09)***	1.05 (1.04,1.07)***
Hemoglobin A1c (%)	1.52 (1.24,1.87)***	1.73 (1.39,2.17)***

*p-value< 0.05; ***p-value< 0.001

**Fig. 1:**
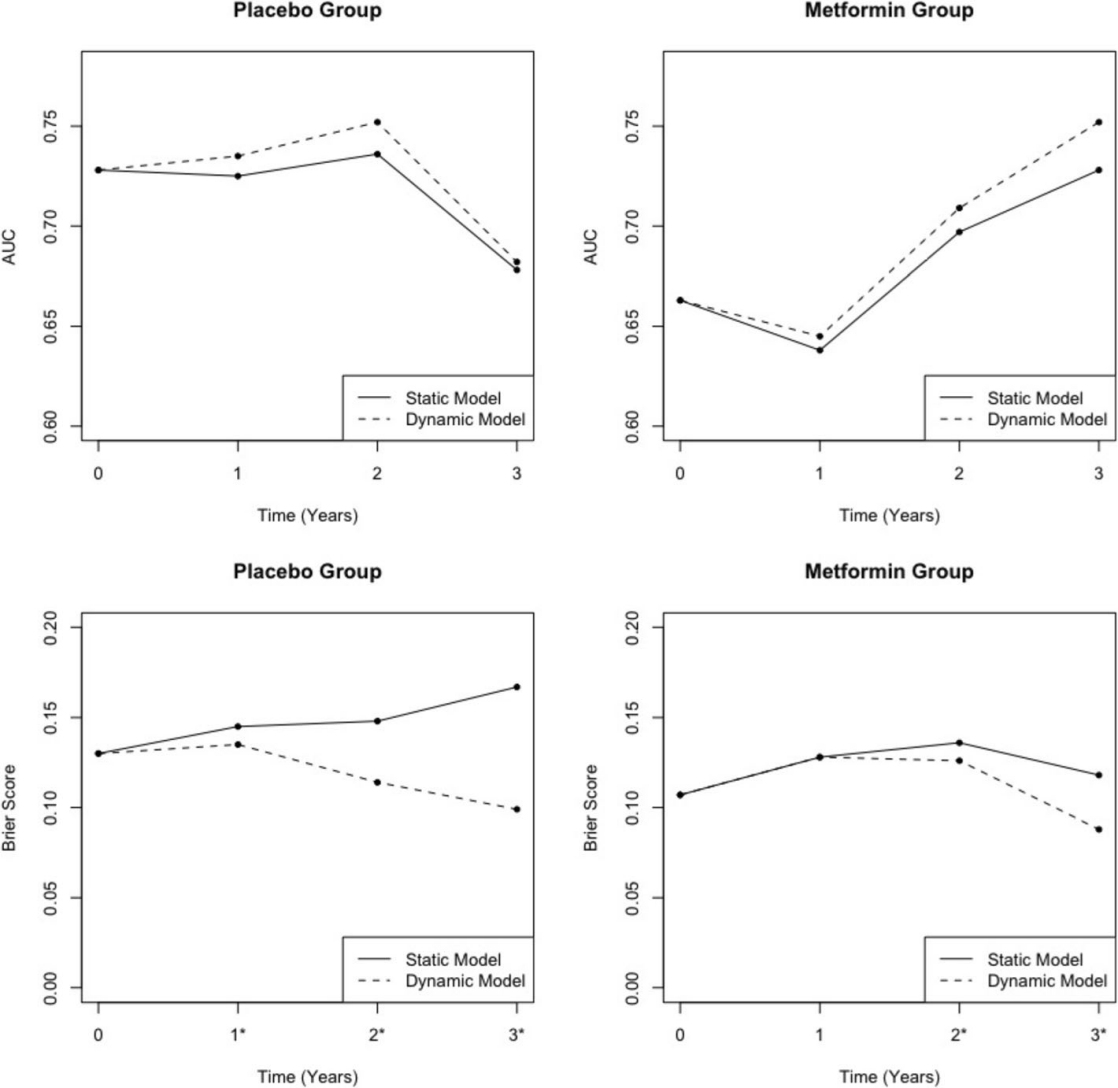
Estimated Area Under the ROC curve (AUC) and Brier Score for Both Prediction Approaches. Note: Higher values for AUC indicate better prediction accuracy. Lower values for the Brier Score indicate better prediction accuracy; *indicates that the two values at this point are significantly different at the 0.05 level i.e., the 95% bootstrap confidence interval for the differences between these two points does not contain zero

In terms of prediction accuracy, at baseline, the static and dynamic models are equivalent and thus, had equal AUC estimates as expected (0.728 for the placebo group and 0.663 for the metformin group). At each subsequent landmark time (years 1, 2, and 3), the AUC of the dynamic model was slightly better than that of the static model (Fig. 1), though not significantly. In the placebo group, the AUC was 0.725 for the static model versus 0.735 for the dynamic model at 1 year (difference 0.010; 95% CI, − 0.015 to 0.035), 0.736 versus 0.752 at 2 years (0.016; − 0.020 to 0.052), and 0.678 versus 0.682 at 3 years (0.004; − 0.043 to 0.051). In the metformin group, the AUC was 0.638 for the static model versus 0.645 for the dynamic model at 1 year (difference 0.007; 95% CI, − 0.027 to 0.041), 0.697 versus 0.709 at 2 years (0.012; − 0.023 to 0.047), and 0.728 versus 0.752 at 3 years (0.024; − 0.029 to 0.077). None of these differences in AUC were statistically significant.

The Brier Score at baseline was 0.130 for the placebo group and 0.107 for the metformin group for both models. At each landmark time, the Brier Score of the dynamic model was lower (i.e., better) than that of the static model (Fig. 1). In the placebo group, these Brier Score differences were statistically significant at all 3 landmark times: 0.145 for the static model versus 0.135 for the dynamic model at 1 year (difference − 0.010; 95% CI, − 0.017 to − 0.003), 0.148 versus 0.114 at 2 years (− 0.034; − 0.044 to − 0.024), and 0.167 versus 0.099 at 3 years (− 0.068; − 0.083 to − 0.053). In the metformin arm, Brier Score differences were statistically significant at 2 years (0.136 static versus 0.126 dynamic; difference − 0.01; − 0.017 to − 0.003) and 3 years (0.118 versus 0.088; − 0.030; − 0.040 to − 0.020).

The Hosmer-Lemeshow test statistics, provided in Table 3, show that for most time points, both the static model and dynamic model are reasonable. There are two exceptions for the static model: when examining the predictions at 3 years in the placebo group, and 1 year in the metformin group where the Hosmer-Lemeshow test statistic indicates significantly poor calibration. For all time points and both groups, the Hosmer-Lemeshow test statistic was lower for the dynamic model when compared to the static model, indicating better calibration as measured by this quantity.

**Table 3:** Hosmer-Lemeshow test statistics

	Static Model		Dynamic Model	
	Hosmer-Lemeshow test statistic	*p*-value^a^	Hosmer-Lemeshow test statistic	*p*-value^a^
Placebo				
Baseline	7.43	0.11	7.43	0.11
1 year	7.28	0.12	5.64	0.23
2 years	5.70	0.22	5.65	0.23
3 years	11.03	0.03	7.95	0.09
Metformin				
Baseline	6.34	0.17	6.34	0.17
1 year	16.40	0.002	7.80	0.10
2 years	7.79	0.10	6.34	0.18
3 years	6.25	0.18	5.68	0.22

^a^*p*-value calculated using chi-squared distribution with degrees of freedom = g-1 = 4 where g = 5 is the number of strata used in the calculation of the Hosmer-Lemeshow test statistic

NRI estimates as well as individual components of this quantity are shown in Table 4. Here, these quantities reflect the extent to which the dynamic landmark model moves an individual’s predicted risk “up” or “down” in the correct direction, compared to the static model. In the metformin group, examining predictions at 1 year, these results show that among those individuals that will have an event within 2 years, the dynamic landmark model gave 40.4% of them a higher risk (correct direction of risk change) and 59.6% a lower risk (incorrect direction of risk change), compared to the static model. Among those that will not have an event within 2 years, the dynamic landmark model gave 38.1% a higher risk (incorrect direction of risk change) and 61.9% (correct direction of risk change) a lower risk. On net, 4.6% of participants had more accurate risk estimates under the dynamic model than under the static model at year 1 (NRI = 4.6, 95% CI: − 15.8 to 24.9%, *p* = 0.661). With the exception of predictions calculated at 1 year in the placebo group, the dynamic model tended to produce more accurate risk estimates than the static model, though these improvements were not statistically significant.

**Table 4:** Net reclassification improvement^a^

Placebo			
	Percentage of individuals for whom the dynamic landmark model estimates a *higher* risk than the static model	Percentage of individuals for whom dynamic landmark model estimates a *lower* risk than the static model	Overall Net reclassification improvement (95% Confidence Interval)
1 year			
Events	**26.5%**	73.5%	−3.8% (−26.0, 18.4%)^b^
Non-events	28.4%	**71.6%**	
2 years			
Events	**4.3%**	95.7%	3.5% (−10.4, 17.3%)
Non-events	2.6%	**97.4%**	
3 years			
Events	**1.4%**	98.6%	1.9% (−7.3, 11.0%)
Non-events	0.4%	**99.6%**	
Metformin			
	Percentage of individuals for whom the dynamic landmark model estimates a higher risk than the static model	Percentage of individuals for whom dynamic landmark model estimates a lower risk than the static model	Overall Net reclassification improvement (95% Confidence Interval)
1 year			
Events	**40.4%**	59.6%	4.6% (−15.8, 24.9%)
Non-events	38.1%	**61.9%**	
2 years			
Events	**19.9%**	80.1%	18.6% (−5.1, 42.4%)
Non-events	10.6%	**89.4%**	
3 years			
Events	**5.0%**	95.0%	7.0% (−12.9, 26.9%)
Non-events	1.5%	**98.5%**	

^a^ Bolding indicates correct risk movement by the dynamic landmark model e.g. individuals who have an event should be given a higher risk

^b^ This calculation is based on: (26.5–73.5) – (28.4–71.6) = −3.8

## Discussion

Our results demonstrate the potential to improve individual risk prediction accuracy by incorporating information about biomarker changes over time into a dynamic modeling approach. Using DPP clinical trial data, we found that incorporating changes in fasting plasma glucose and HbA1c into the diabetes prediction model moderately improved predication accuracy, in terms of calibration, among study participants in both the placebo and metformin trial arms.

However, we found no evidence of improvements in terms of discrimination (i.e, AUC or NRI) when the dynamic model was used. This is not unexpected given that calibration and discrimination each measure important, but distinct, aspects of prediction accuracy [43, 44]. These results indicate that while the dynamic model does not appear to significantly improve the ordering or ranking of individuals in terms of risk of a diabetes diagnosis, the approach does improve upon the absolute risk estimates compared to the static model. The clinical significance of this improvement in accuracy as measured by the Brier Score and the Hosmer-Lemeshow test statistic depends on the practical use of the calculated predictions. For example, if risk estimates are to be compared to certain absolute thresholds for the purpose of clinical decision making—for example, when an intervention or treatment will be initiated if the risk of an event exceeds 10% - our observed small but significant improvement in precision may be considered clinically meaningful. However, the additional computational complexity required to implement the dynamic prediction model may not be worth the trade-off for this small improvement.

The methodology described here offers a straightforward approach to developing more accurate and personalized prediction rules for individual patients. In addition, this approach can be extended to take advantage of longitudinal electronic health record data that might already be available in practice. Multiple areas of health research have focused on collecting and improving the utility of a vast amount of patient-level data, for example, by allowing for data collection using smartphones or tablets [45, 46]. The development of methods that can use this wealth of data to appropriately inform decision-making warrants further research. While most risk predictions are based on static models, there are some notable exceptions that have been developed very recently such as the Million Hearts Longitudinal Atherosclerotic Cardiovascular Disease Risk Assessment Tool [47] which uses a dynamic prediction modeling approach.

Though we do not focus heavily here on discussing the estimated association between covariates and the primary outcome (i.e., the model coefficients and hazard ratios), we have assumed that these associations would be important to practitioners in this setting. For example, both practitioners and patients may wish to view explicit regression coefficients to understand the contribution of each risk factor to their risk score [48]. If this were not the case, and only the individual predictions were needed, then other approaches, such as machine learning approaches including boosting algorithms and artificial neural networks -- which could incorporate this dynamic prediction concept-- should also be considered [49–52]. Though these approaches do not provide explicit estimates of associations between individual covariates and the primary outcome (e.g. regression coefficient estimates), they might be useful when relationships between covariates and primary outcomes are complex (e.g. nonlinear, nonadditive, etc.), and/or a large number of covariates is available (e.g. genetic information). Future research comparing our approach to machine learning approaches in a dynamic prediction framework is warranted.

Our study applying these methods to the DPP data has some limitations. First, since these data are from a clinical trial that was specifically focused on high-risk adults, these results may not be representative of individuals at lower risk for diabetes. Second, our data lacked precise information on patient characteristics (exact age and BMI, for example) and was limited to the biological information available in the DPP data release. This may have contributed to our observed overall moderate prediction accuracy even using the dynamic model in the 0.6–0.7 range for the AUC. Future work examining the utility of dynamic models is warranted within studies that have more patient characteristics available for prediction. However, even with this limitation, this illustration shows the potential advantages of such a dynamic approach over a static approach.

## Conclusions

Dynamic prediction has the potential to improve the accuracy of future health status predictions for individual patients. Given the widespread use of risk prediction tools in population management and clinical decision making, even modest enhancements in prediction accuracy could yield improvements in care for large numbers of patients—at little added cost or effort.

## Additional file

Additional file 1: **Table S1** Dynamic Prediction Model, Metformin Group. **Table S2** Dynamic Prediction Model, Placebo Group. (DOCX 27 kb)

## Data Availability

DPP data are publicly available upon request from the NIDDK Data Repository and require the establishment of a data use agreement: https://repository.niddk.nih.gov/home/.

## Abbreviations

AUC: Area under the receiver operating characteristic curve
BMI: Body mass index
CI: Confidence interval
DPP: Diabetes Prevention Program
HbA1C: Hemoglobin A1c
NIDDK: National Institute of Diabetes and Digestive and Kidney Diseases
NRI: Net reclassification index
