# Supplementary material for: Dynamic risk prediction for diabetes using biomarker change measurements
Source: BMC Med Res Methodol. 2019 Aug 14;19:175. doi: 10.1186/s12874-019-0812-y (PMC6694545; doi:10.1186/s12874-019-0812-y)
Supplement: Supplementary file 1 — Table S1 Dynamic Prediction Model, Metformin Group. Table S2 Dynamic Prediction Model, Placebo Group. (DOCX 27 kb) [file 12874_2019_812_MOESM1_ESM.docx]

**Additional file 1**

**Table S1 Dynamic Prediction Model, Metformin Group**

|  | Meformin  [prediction at 1 year post-baseline]  Hazard Ratio (95% Confidence Interval) | Meformin  [prediction at 2 years post-baseline]  Hazard Ratio (95% Confidence Interval) | Meformin  [prediction at 3 years post-baseline]  Hazard Ratio (95% Confidence Interval) |
| --- | --- | --- | --- |
| Age |  |  |  |
| <40 | REF | REF | REF |
| 40-44 | 1.07(0.71,1.6) | 0.91(0.59,1.42) | 0.87(0.52,1.48) |
| 45-49 | 0.78(0.52,1.16) | 0.58(0.37,0.89)* | 0.55(0.32,0.93)* |
| 50-54 | 0.9(0.62,1.31) | 0.74(0.49,1.12) | 0.81(0.5,1.32) |
| 55-59 | 0.76(0.49,1.19) | 0.65(0.39,1.06) | 0.56(0.31,1.03) |
| 60-64 | 1.03(0.66,1.59) | 0.92(0.58,1.48) | 0.86(0.49,1.52) |
| 65+ | 0.76(0.47,1.23) | 0.66(0.38,1.14) | 0.61(0.32,1.16) |
| Gender |  |  |  |
| Male | REF | REF | REF |
| Female | 1.09(0.85,1.4) | 1.08(0.81,1.43) | 1.35(0.96,1.9) |
| BMI |  |  |  |
| <30 kg/m^2^ | REF | REF | REF |
| ≥30 to < 35 kg/m^2^ | 1.07(0.8,1.43) | 1.16(0.83,1.63) | 1.1(0.75,1.61) |
| ≥35 kg/m^2^ | 1.2(0.89,1.61) | 1.51(1.08,2.1)* | 1.24(0.85,1.82) |
| Smoking Status |  |  |  |
| Yes | 1.29(0.84,1.97) | 1.14(0.69,1.89) | 0.75(0.37,1.5) |
| No | REF | REF | REF |
| Not available | 1.42(0.96,2.12) | 1.35(0.85,2.12) | 1.82(1.1,3)* |
| Race/ethnicity |  |  |  |
| White | REF | REF | REF |
| Black | 1.16(0.87,1.54) | 1.37(1.01,1.86)* | 1.26(0.87,1.82) |
| Hispanic | 0.95(0.69,1.32) | 0.93(0.65,1.35) | 0.91(0.59,1.4) |
| Other | 0.88(0.48,1.61) | 1.2(0.64,2.26) | 1.13(0.51,2.47) |
| Fasting plasma glucose at baseline (mg/dL) | 1.07(1.05,1.08)*** | 1.08(1.06,1.1)*** | 1.08(1.06,1.1)*** |
| Hemoglobin A1c at baseline (%) | 1.52(1.15,2.01)** | 1.49(1.1,2.02)** | 1.71(1.17,2.51)** |
| Change in fasting plasma glucose from baseline to prediction year [1,2, or 3] (mg/dL) | 1.03(1.01,1.04)*** | 1.03(1.02,1.04)*** | 1.03(1.01,1.05)*** |
| Change in hemoglobin A1c from baseline to prediction year [1,2, or 3] (%) | 0.93(0.6,1.42) | 1.3(0.84,2) | 1.82(1.13,2.94)* |

**Table S2 Dynamic Prediction Model, Placebo Group**

|  | Placebo  [prediction at 1 year post-baseline]  Hazard Ratio (95% Confidence Interval) | Placebo  [prediction at 2 years post-baseline]  Hazard Ratio (95% Confidence Interval) | Placebo  [prediction at 3 years post-baseline]  Hazard Ratio (95% Confidence Interval) |
| --- | --- | --- | --- |
| Age |  |  |  |
| <40 | REF | REF | REF |
| 40-44 | 0.95(0.64,1.4) | 0.96(0.61,1.52) | 0.73(0.42,1.26) |
| 45-49 | 0.96(0.68,1.35) | 0.86(0.58,1.29) | 0.74(0.46,1.16) |
| 50-54 | 0.77(0.53,1.14) | 0.77(0.49,1.2) | 0.8(0.49,1.31) |
| 55-59 | 0.62(0.4,0.94)* | 0.46(0.27,0.77)** | 0.45(0.26,0.8)** |
| 60-64 | 0.61(0.37,1)* | 0.62(0.35,1.08) | 0.59(0.32,1.11) |
| 65+ | 0.74(0.45,1.22) | 0.7(0.4,1.25) | 0.61(0.32,1.19) |
| Gender |  |  |  |
| Male | REF | REF | REF |
| Female | 1.19(0.93,1.52) | 1.32(0.99,1.76) | 1.3(0.93,1.83) |
| BMI |  |  |  |
| <30 kg/m^2^ | REF | REF | REF |
| ≥30 to < 35 kg/m^2^ | 1.04(0.78,1.37) | 1.04(0.76,1.44) | 1.12(0.77,1.63) |
| ≥35 kg/m^2^ | 1.12(0.85,1.48) | 0.97(0.7,1.34) | 1.05(0.72,1.52) |
| Smoking Status |  |  |  |
| Yes | 1.1(0.76,1.6) | 1.29(0.84,2) | 1.43(0.86,2.39) |
| No | REF | REF | REF |
| Not available | 1.23(0.81,1.88) | 1.09(0.67,1.77) | 1.19(0.69,2.07) |
| Race/ethnicity |  |  |  |
| White | REF | REF | REF |
| Black | 1.4(1.06,1.84)* | 1.84(1.34,2.53)*** | 1.75(1.21,2.54)** |
| Hispanic | 1.25(0.91,1.71) | 1.23(0.85,1.8) | 1.21(0.79,1.85) |
| Other | 1.57(1,2.48) | 1.89(1.12,3.19)* | 1.95(1.05,3.61)* |
| Fasting plasma glucose at baseline (mg/dL) | 1.09(1.07,1.11)*** | 1.1(1.08,1.13)*** | 1.09(1.06,1.11)*** |
| Hemoglobin A1c at baseline (%) | 1.32(1.01,1.73)* | 1.01(0.73,1.38) | 0.99(0.69,1.43) |
| Change in fasting plasma glucose from baseline to prediction year [1,2, or 3] (mg/dL) | 1.03(1.02,1.05)*** | 1.06(1.04,1.08)*** | 1.06(1.04,1.08)*** |
| Change in hemoglobin A1c from baseline to prediction year [1,2, or 3] (%) | 1.22(0.83,1.78) | 1.16(0.77,1.73) | 1.87(1.16,3)** |
